# Hoof Matters: Developing an Athletic Thoroughbred Hoof

**DOI:** 10.3390/ani12223119

**Published:** 2022-11-11

**Authors:** Sarah Jane Hobbs, Simon Curtis, Jaime Martin, Jonathan Sinclair, Hilary M. Clayton

**Affiliations:** 1 Research Centre for Applied Sport, Physical Activity and Performance, University of Central Lancashire, Preston PR1 2HE, UK; 2 Church Cottage, 59 The Street, Newmarket IP28 6PL, UK; 3 School of Animal Rural & Environmental Sciences, Nottingham Trent University, Nottingham NG1 4FQ, UK; 4 Sport Horse Science, LLC, Mason, MI 48854, USA

**Keywords:** foal, hoof, hoof angle, hoof development, hoof growth, hoof deformation

## Abstract

**Simple Summary:**

The development of strong, well-conformed hooves begins pre-natally and is on-going throughout the horse’s life. This paper explores specific aspects of the development and adaptation of the distal forelimb in Thoroughbred foals with emphasis on how the hoof responds to weight bearing early in life. The thickness of the epidermal tissues at the toe increased from 2.84 ± 0.41 mm before birth to 4.04 ± 1.10 mm by 4 months of age. The increase in thickness was accompanied by decreased density of horn tubules at the toe while the number and size of horn tubules increased at the medial and lateral quarters. This provides a malleable hoof capsule with increased resistance to fracture toughness at the quarters to allow for skeletal growth. Between 4–6 months of age, the hoof widens, and higher loading on the medial side (>60%) vs. the lateral side (<40%) may be factors that influence mature asymmetric hoof shape. Shortly after 12 months-of-age, the dorsal hoof wall angle becomes parallel with the dorsal wall of the distal phalanx, thus optimizing the functional capacity of the hoof capsule in the weanling Thoroughbred.

**Abstract:**

Conformation of the hooves and distal limbs of foals and factors influencing their morphological development have not been reported in detail for the Thoroughbred breed. In this paper we explore morphogenesis of the equine distal limb in Thoroughbred foals with emphasis on adaptations in response to weight bearing early in life that prepare the foal for an athletic career. Novel data from four studies are presented chronologically during key time periods to illustrate specific aspects of distal limb growth and adaptation. Dorsal epidermal thickness increased from 2.84 ± 0.41 mm in utero to 4.04 ± 1.10 mm by 4 months of age. The increase in thickness was accompanied by decreased tubular density, increased inter-tubular material, and an increase in number and size of tubules at the quarters, which provided a malleable hoof capsule to allow for skeletal growth. Between 4–6 months of age, the hoof widens, and higher loading on the medial side (>60%) vs. the lateral side (<40%) may be factors that influence mature asymmetric hoof shape. Shortly after 12 months-of-age, the dorsal hoof wall angle and dorsal parietal angle of the distal phalanx become parallel, thus optimizing the functional capacity of the hoof capsule in the weanling Thoroughbred.

## 1. Introduction

Horses belong to the taxonomic order Perissodactyla which is distinguished by having an odd number of toes; in horses a single weight-bearing toe is derived from the third digit. Furthermore, horses are unguligrade implying that the metapodials and digits are elevated from the ground. As a result, weight-bearing has become the responsibility of the tip of the third digit encased by the modified nail or ungula. The hoof consists of an outer protective layer called the hoof capsule derived from the nail and composed of insensitive cornified tissues. Inside the hoof capsule are sensitive tissues including bone, ligament and tendon that are part of the locomotor apparatus.

The horse’s outer appearance or conformation is aesthetically and functionally important with particular aspects of conformation being prioritized in specific breeds. Correct digital alignment and conformation are emphasized in Thoroughbred racehorses because the high speeds at which they train and race are associated with high impact accelerations [1,2] and exceptionally large ground reaction forces, especially in the forelimbs [3]. Mawdsley et al. [4] described large phenotypic variation among superior 2–3 year-old Thoroughbred racehorses and proposed that static conformational assessments performed in conjunction with dynamic assessments would provide useful selection criteria.

In the sagittal plane, the hoof angle, measured mid-dorsally at the toe should equate to the pastern axis. The resulting hoof-pastern axis is regarded as a crucial conformational feature [5] with regard to optimizing forces in the distal limb [6] and reducing the risk of injuries [7]. Although forelimb alignment has been studied quite extensively in the frontal plane [8,9], there is less information describing sagittal plane digital angles and how they change during the first year of life in the Thoroughbred. Defective distal limb conformation, such as a misaligned hoof-pastern axis, was not found to limit performance [10] but may have a negative effect on the longevity of a Thoroughbred’s racing career. This, in turn, has financial implications for the breeding and racing industries.

The role of the hoof in the hoof-surface interaction of the galloping horse includes assisting in damping impact accelerations and high magnitude ground reaction forces. This is achieved by converting impact energy into strain energy, supporting the limb during maximal loading and providing grip [11]. The structural composition and material properties of a healthy hoof wall, together with intricate design features to reduce stress at the interface between the hoof wall and skeleton allow for such demanding functional capacity. As an anisotropic viscoelastic composite reinforced by multi-directional fibres [12,13,14,15,16], the hoof is mechanically stable and has been measured to deform at the heels by up to almost 16 mm when galloping at 12 m/s [17].

The hoof wall consists of 3 layers: the stratum externum consisting of the thin layer of periople, the stratum medium characterized by its tubular and inter-tubular horn structure, and the stratum internum with its inwardly projecting lamellae that interdigitate with corresponding dermal lamellae from the distal phalanx [18]. The stratum medium is the thickest of the 3 layers and is responsible for supporting the weight of the horse and for transferring ground reaction forces generated when the hoof presses against the ground to the appendicular skeleton [12]. This interface allows for a graduated stress distribution due to the higher moisture content and consequently greater flexibility of the inner part of the hoof wall that is closest to the sensitive structures [14,19]. In the athletic horse, the parallel arrangement between the dorsal hoof wall and the dorsal parietal surface of the distal phalanx is essential for optimal functional capacity.

The strong rigid hooves that are necessary for supporting body weight during locomotion pose a risk of damage to the intra-uterine tissues during gestation and to the birth canal during parturition. To reduce the risk of injuring the mare, the fetus develops soft, protective ‘slippers’ that form a gelatinous deciduous hoof capsule [20] that protects the maternal tissues from injury when the foal is in utero and during parturition. Foals are precocial and can usually walk, trot and canter within one to two hours after birth. This calls for rapid shedding of the soft, deformable, fetal hoof capsule and its transformation into a weight-bearing structure. Together with support from muscles, tendons, and ligaments that support tensile forces acting across the joints and maintain functional limb alignment, the foal can very quickly maintain upright posture. Over the following weeks and months of a foal’s life, the shape and size of the hoof capsule and its relationship with the internal hoof structures is modified as the mature morphology is established [21,22]. The keratinized hoof capsule continues to grow in a proximodistal direction throughout life in accordance with internal and external forces. Frequent and regular hoof trimming is required to maintain the correct hoof shape and balance, especially in the early months of life [22].

Conformational differences between foals and mature horses are evident in the external features of the hoof and underlying skeletal structures. However, limited information has been reported to date on factors that influence hoof growth and development either in Thoroughbreds or other breeds. Four studies were carried out by Curtis [23] to document aspects of growth and development and factors that influence hoof shape in Thoroughbreds foals, from late gestation to weanling age. In this paper we investigate morphogenesis of the equine distal limb with emphasis on adaptative responses to weight bearing early in life that prepare the foal for an athletic career. Novel data from Curtis [23] are evaluated chronologically in the life of the foal to illustrate specific aspects of the growth and adaptation of the hoof capsule and digital conformation in young Thoroughbreds.

## 2. Methods

Four independent studies were conducted to evidence changes in hoof shape in this population using convenient sampling of Thoroughbred fetuses and foals ranging from 38 days pre-partum to 503 days post-partum.

### 2.1. Measurement of Epidermal Features

The subjects were 15 Thoroughbred cadaver fetuses and foals, aged −38 to 134 days, that had died naturally or were still born, none had been euthanized. All subjects had healthy limbs and hooves. After disarticulating the digits at the metacarpophalangeal joint, they were frozen within 2 h after death.

The hooves were sectioned parallel to the coronary band, midway between the coronary band and the bearing border. Tissue samples were collected from the hoof wall on the dorsal, lateral and medial sides, as described by Reilly et al. [24]. The samples were preserved in 10% buffered neutral formalin, embedded in blocks of paraffin and softened with a hair removal product. A bench microtome was used to cut 4µm sections for preparing slides. After being stained with haematoxylin and eosin, a Nikon Eclipse 50i microscope equipped with a camera was used to visualize the slides and record images at magnifications of ×200 and ×400 with an embedded scale for calibration. The images were saved as lossless Jpeg files. A Neubauer haemocytometer was used to check the calibration and found 99% precision.

**Figure 1:**
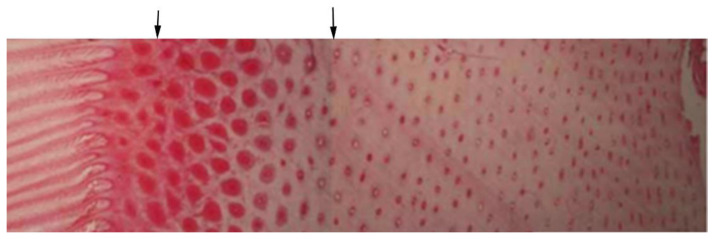
Three microscopic images matched and concatenated at the positions indicated by the black arrows to construct a single image of the hoof wall from the lamellae (**left**) to the outer wall (**right**).

Two parallel lines, 1 mm apart, were added to each image using Photoshop 7.03 (Adobe, San Jose, CA, USA). A single image showing the full width of the hoof wall was created by concatenating sequential images (Figure 1). Epidermal thickness was measured with three repetitions and the mean value recorded. The three tubules with the largest diameters were identified subjectively [25], the diameter of each tubule was measured three times, and the mean of the 9 measurements was recorded. The number of horn tubules in which the whole of the medulla was visible was counted using ImageJ (National Institute of Mental Health, Bethesda, MD, USA). Foal identification, age, epidermal thickness, largest tubule diameter and number of tubules were tabulated in Excel 2007 (Microsoft Corp., Redmond, WA, USA) for the lateral, medial and dorsal regions of the hoof wall. Tubule density was the tubule number divided by the measured area (tubules/mm^2^).

### 2.2. Measurement of Skeletal Conformational Features

Twenty-two Thoroughbred foals that were born during the same year (2011) at three stud farms located in close proximity and using the same farrier and veterinary practice. None of the foals had obvious conformational faults or had been treated for lameness and all foals were handled daily by experienced handlers. During data collection, the foals and their dams were controlled by experienced handlers. They were habituated to and monitored throughout the measurement procedures to ensure that no undue stress was caused. An experienced radiographer collected and processed the radiographic images using standard procedures. All foals were measured on the same day in the months of June when they were 33 to 145 days old and November when they were 180 to 296 days old. The reasons for selecting data from these months were that in June the foals were at least a month old and after November some foals would be sold and not available to include in the study. All of the foals’ had their hooves trimmed by the same farrier according to a published protocol [26] and none were trimmed during the 2 weeks preceding data collection.

A line was drawn in indelible ink through the middle of the frog to the point of toe to establish the midpoint of the dorsal wall [24]. The solear length of each hoof was measured three times with a flexible ruler from the toe through the frog apex to the point of buttress [27]. Using the mark in the middle of the toes as a guide, a radio-opaque disc was fixed mid-dorsally on the hoof wall distal to the coronet using adhesive tape. This disc was used for linear calibration of the radiographic image.

**Figure 2:**
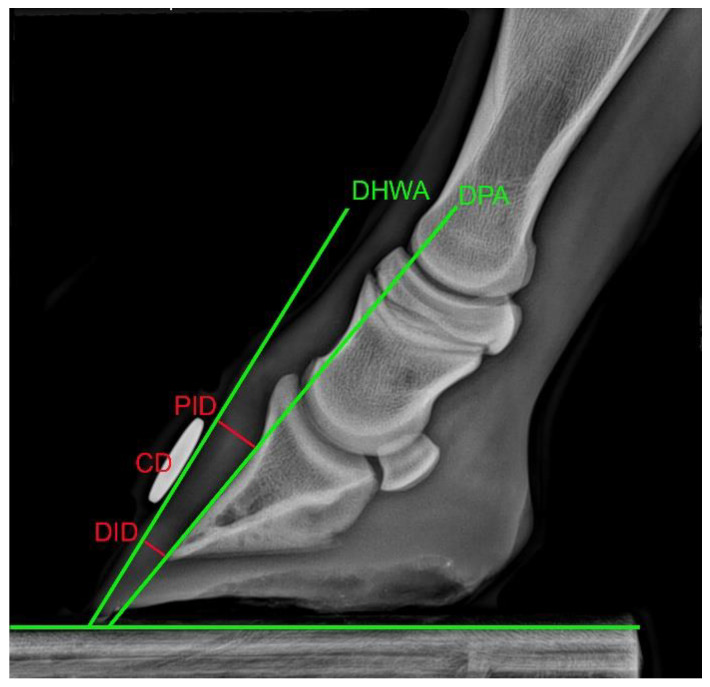
Lateromedial radiographic view of the left front digit of an 8-week-old foal. Angular measurements made relative to the horizontal are marked by green lines and linear measurements are shown by red lines. DHWA: dorsal hoof wall angle; DPA: dorsal parietal angle; PID: proximal integument depth; DID: distal integument depth; CD: disc used to calibrate measurements of PID and DID.

The foals’ fore hooves were then placed upon two wooden blocks, 50 mm high, on a level floor with the metacarpal segments vertically aligned [28]. Standard lateromedial (LM) radiographic views were taken with a mobile radiography unit (Sound-Eklin TuDR, BCF Technology Ltd., Livingston, UK) and saved in Dicom format. The resulting images were used to measure the dorsal hoof wall angle (DHWA), the dorsal parietal angle of the distal phalanx and the integument depth of the dorsal hoof wall with OsiriX 3 (OsiriX Technologies Inc., Marietta, GA, USA) with an accuracy of <0.1 ± 0.03 mm (mean ± standard error) (Figure 2). The proximal integument depth (PID) was measured from the concave curvature of the distal phalanx (PIII) to the abaxial extent of the dorsal wall at 90° to the dorsal hoof wall angle (DHWA). The distal integument depth (DID) was measured from the distal tip of PIII to the abaxial extent of the dorsal wall at 90° to the DHWA. The dorsal parietal angle (DPA) was defined by an angle connecting the PID and the DID. Each measurement was taken three times from all images and transferred into an Excel spread sheet where the mean was pooled.

### 2.3. Measurement of Growth and Compression

Twenty-eight Thoroughbred foals born in 2013 (age 13 to 77 days at the start of the study) were selected from two stud farms on the basis of being healthy and having no behavioral difficulties. For these foals, data were collected when acquired flexural deformities (AFDs) are reported to emerge. Foals included in this study were categorized visually according to the presence or absence of AFD. Foals in the AFD group were treated by trimming the heels, complete stable rest for up to 2 weeks and, in two cases, a toe extension was applied. An additional twelve Thoroughbred weanlings born in 2013 (285 ± 26 days at the start of the study) were selected to extend the hoof growth and compression dataset with the following criteria; healthy foals born at two stud farms during the same year, that had no history of lameness. The management involved daytime turnout on pasture with overnight stabling and supplementary feeding of a well-balanced diet. They were handled by experienced personnel at all times, including during data collections. Immediately prior to each measurement, the same farrier trimmed the feet and rounded the distal border to a radius of approximately 3 mm without rasping the outer hoof wall.

**Figure 3:**
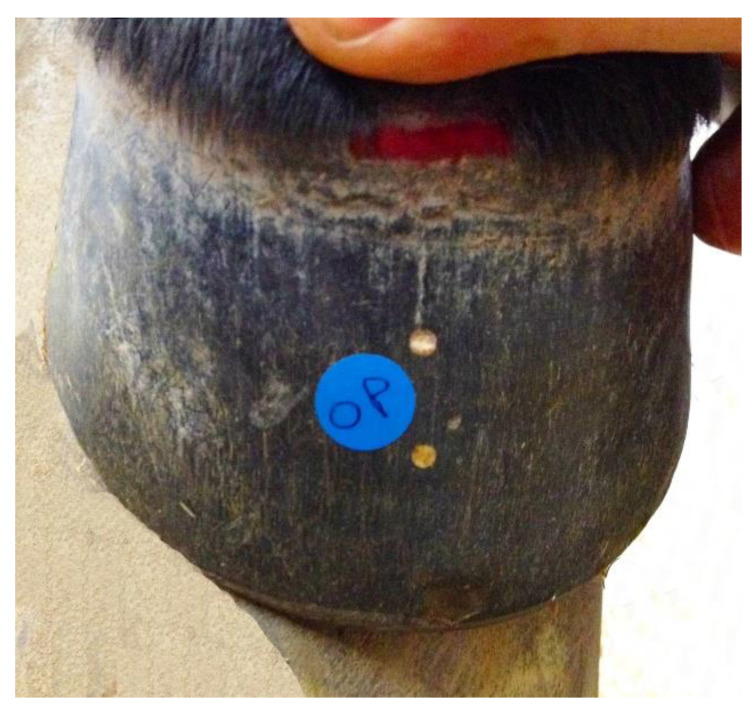
Dorsal image of a foal hoof showing the two points of acrylic and the disc used to calibrate growth and compression of the dorsal hoof wall. The hair has been raised to show the coronary hairline.

The coronary hairline was defined with red indelible ink. The mid-dorsal centre of the hoof wall at the bearing surface was located as described in Section 2.2 and a line was drawn dorsally from the bearing surface to the coronary hairline. Along this line, two indentations were made approximately 15 mm and 30 mm below the coronary hairline. The coronary hairline and two indentations following the line of the tubules were also made at the widest part of the foot (quarters). The indentations were filled with colored acrylic and allowed to set. After the acrylic had hardened, it was filed level with the hoof wall creating two easily visible reference points. A 12.7 mm circular disc was attached to the hoof wall between the two acrylic reference points for calibration purposes (Figure 3).

With the hoof lifted and extended, five photographs were taken using a digital camera with the lens perpendicular to the dorsal hoof wall from a distance of 0.3 m. Lateral and medial photographs were taken in a similar manner. The photographs were observed immediately to check they were correctly framed and focused. The best image was selected and downloaded to a software package (Metron Hoof 5.31, Epona Tech, Creston, IA, USA) designed to extract hoof measurements [29]. Repeat photographs were taken every four-weeks the distal mark was no longer visible due to being removed by wear or trimming at the bearing border. For weanlings, only dorsal measurements were taken.

Linear dimensions of the hoof were measured three times each using the circular disc for calibration and the data were recorded in Excel. Toe length was measured between the coronary hairline and bearing border. The distance from the coronary hairline to the proximal acrylic reference point was measured to determine hoof growth and hoof growth was divided by the number of days since the previous evaluation to calculate hoof growth rate. The distance between the two acrylic points at the first data collection was used as a reference for measuring hoof compression. Compression rate was calculated as the amount of compression divided by the number of days between measurements. From these measurements toe length, hoof growth rate and hoof compression rate were determined at each data collection.

### 2.4. Measurement of Solear Load Distribution

The 28 Thoroughbred foals selected for growth and compression measurements were also used to measure solear load distribution. After the fore hoof photographs had been taken, they were walked onto a pressure mat which recorded the loading pattern at 50 Hz for 20 s during standing. No attempt was made to move or alter the foals’ stance after they stopped on the pressure mat. Three recordings were made and saved in Footscan Balance. The recording that showed the smallest amount of sway was chosen for analysis and frame 500 from that recording was used for analysis. An image from this frame was extracted from Footscan Balance and the dorsopalmar axis of each hoof was determined in the horizontal plane using Photoshop 7.03 using the pressure contours of the frog and external hoof features to align the axis.

**Figure 4:**
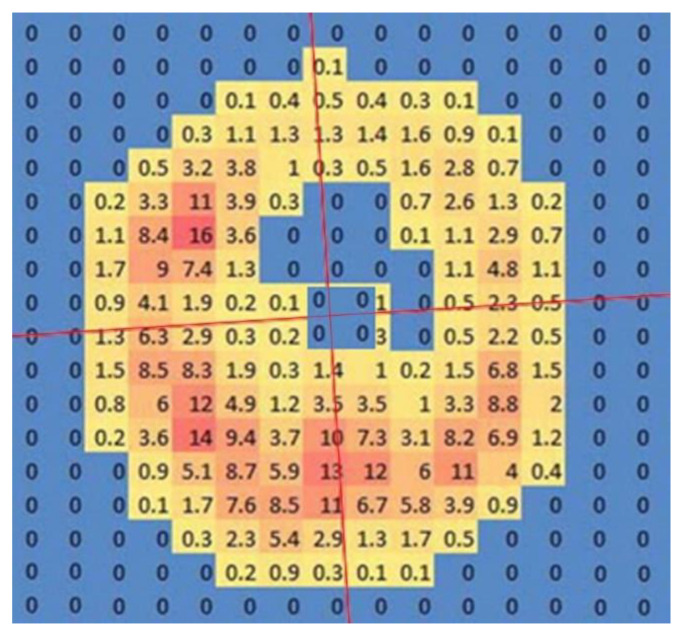
Scan of hoof pressure. The perpendicular red lines indicate the cross hairs intersecting at the geometric centre of the data rotated to align with the dorsopalmar axis of the hoof.

The selected trial was exported to Excel and the chosen frame was used to obtain an image of each loaded cell from which the left and right force values were calculated. Cross hairs positioned at the geometric centre of the data were overlaid on the force data and rotated to align with the hoof’s dorsopalmar axis (Figure 4).

**Figure 5:**
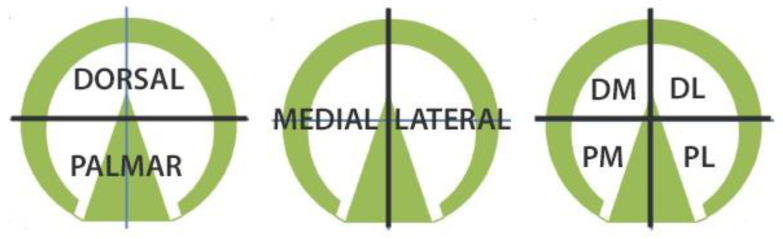
Subdivision of the solear surface of the hoof used to compare force values. (**Left**): transverse division into dorsal and palmar; (**Centre**): median division into medial and lateral; (**Right**): division into quadrants. DM: dorsomedial; DL: dorsolateral; PM: palmaromedial; PL: palmarolateral.

The force values in each (dorsomedial, dorsolateral, palmaromedial, palmarolateral) were calculated by summation of the forces for all loaded cells in the quadrant (Figure 5). Where the cross hair split a cell, the value was shared equally between the two quadrants. Growth and compression for the different heel regions were calculated by combining the forces for two quadrants. Force distribution as percentages was calculated for the dorsal vs. palmar and medial vs. lateral halves.

### 2.5. Statistical Analysis

Data from all four studies were combined into one spreadsheet in SPSS 28.0.1.1 (IBM, Armonk, NY, USA). Study, foal identification, age, limb and measured variables for each study were input. The data were then separated by age, based on the following criteria: −38 days to birth (non-weightbearing), 1 day to 4 months of age (based on the emergence of AFD [30]), 4 to 6 months (based on changes in hoof shape of Arabian foals [31]) and 6 to 12 months (based on hoof shape changes [19,32]). Data from each dataset were analyzed separately in each period where data was available.

For epidermal features, regional differences in structure were compared using a General Linear Model of covariance with foal as a covariate and significance set at *p* < 0.05. Where significant differences were found, Bonferroni post hoc pair-wise comparisons were performed to identify which regions differed significantly.

For skeletal conformational features, descriptive statistics (mean ± standard deviation) were calculated for each available period. In addition, to illustrate the sagittal plane conformational changes over time, the slope of the DHWA and dorsal parietal angle of the distal phalanx, and the slope of the proximal integument depth and distal integument depth were compared for statistical significance (*p* < 0.05).

For growth, compression and loading measurements, foals were grouped into control and AFD groups. Two analyses were performed with these data. Firstly, in the control group only, regional differences in variables were compared using a General Linear Model of covariance with foal as a covariate and significance set at *p* < 0.05. Where significant differences were found, Bonferroni post hoc pair-wise comparisons were performed to identify which regions differed significantly. Secondly, the difference in hoof growth rate, compression rate and load distribution between groups was analyzed using a General Linear Model of covariance with foal as a covariate and significance set at *p* < 0.05. Additional descriptive data are provided for later periods where it was available.

## 3. Results and Discussion

The functional capacity of the hoof in the Thoroughbred racehorse begins with the development of the hoof capsule in utero. Post-partum changes due to growth and in response to loading influence the form and function of the mature hoof.

### 3.1. The Foetal Hoof Capsule

**Figure 6:**
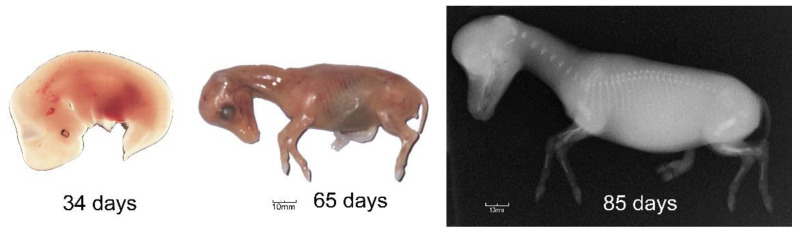
Different stages of embryonic and fetal development. (**Left**): 34-day embryo with left forelimb bud visible; centre: 65 day fetus with developing hooves; (**Right**): radiograph of 85 day fetus showing rudimentary skeleton. Reproduced with permission from S.J. Curtis, The Hoof of the Horse; Newmarket Farrier Consultancy, 2019.

Gestational length in horses averages 341 days and is usually a little longer for males than females [33]. During embryonic development, the limb buds are still undifferentiated at 20 days of gestation but by 27 days hoof formation has become evident and the thoracic limb bud has formed completely by 34 days [34] (Figure 6). The hooves are easily identified at 65 days even though the bones in the distal limb are not visible radiographically until 85 days [35] (Figure 6).

**Figure 7:**
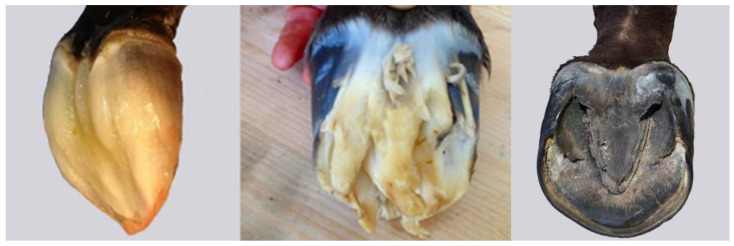
Deciduous hoof capsule forming in a 9-month-old fetus (**left**), fully developed deciduous hoof capsule in a newborn foal prior to standing (**centre**), and solear surface of a foal hoof after one month of weight-bearing (**right**). Reproduced with permission from S.J. Curtis, The Hoof of the Horse; Newmarket Farrier Consultancy, 2019.

In the later part of gestation, the fetal hoof develops a deciduous hoof capsule, sometimes called a ‘slipper’, consisting of soft, gelatinous material that prevents damage to the maternal tissues during late pregnancy and parturition (Figure 7). It is derived primarily from the sole and frog, with contributions from the coronary corium and the hoof wall. The deciduous hoof capsule is replaced continually by the newly forming, cornified permanent hoof capsule [20].

**Figure 8:**
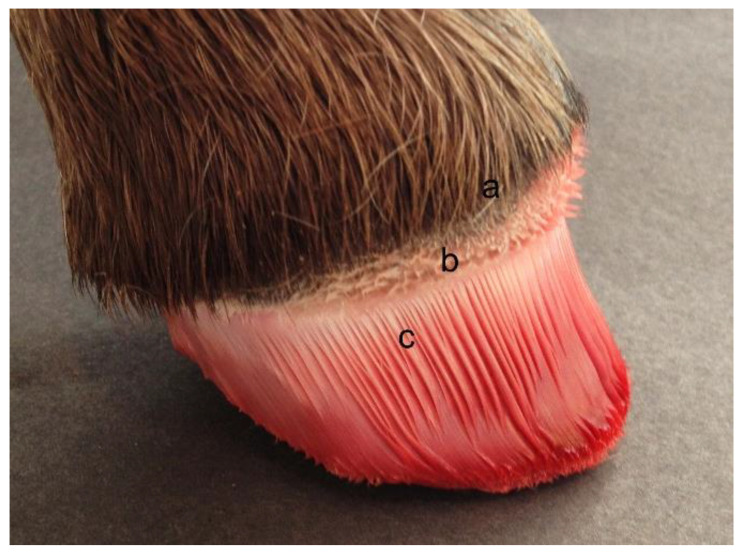
A foal’s foot after removal of the hoof capsule. (a) the coronary hairline; (b) papillae in the coronary band; (c) lamellae. Reproduced with permission from S.J. Curtis, The Hoof of the Horse; Newmarket Farrier Consultancy, 2019.

During fetal development the shape and dimensions of the dermal papillae and lamellae are established. These influence the structure of the cornified hoof capsule and determine the mechanical quality of the hoof horn later in life (Figure 8). Adequate horn quality is needed to fulfil the functions of supporting the hoof capsule, shock absorption, protecting the underlying dermal tissues, and contributing to the suspensory apparatus of the distal phalanx [36].

Our histological evaluations of cadaver fetuses and foals aged −38 to 134 days showed that in the later stages of gestation leading up to birth, the epidermis develops features that are found in the mature horse. The epidermal hoof wall becomes thickest dorsally, and this region has both the largest number of tubules and the largest tubule diameter (Table 1). Horn tubules located more peripherally in the hoof wall are smaller (Figure 1) as described by Leach in the mature horse [25].

Prior to weightbearing, the structure of the fetal hoof is symmetrical between its medial and lateral sides (Table 1). This is in contrast to mature horses, in which the epidermis is thicker medially than laterally [37,38] and tubule density is greater in the medial compared to the lateral region [24,37]. These findings suggest that the development of abaxial asymmetries in the epidermal hoof tissues represent the effects of environmental stimulation on the blank canvas of the neonatal hoof.

### 3.2. The Foal Hoof Capsule (0–4 Months)

**Figure 9:**
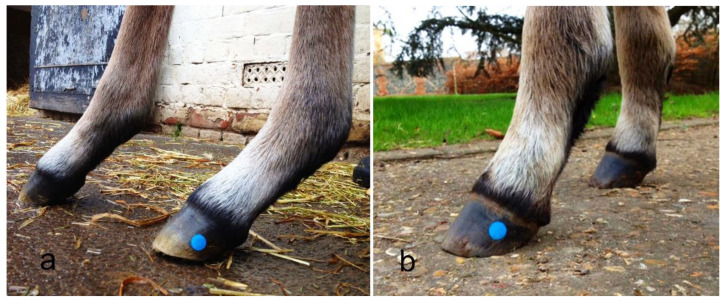
The same foal shown on left at 12 h post-partum and on right at 14 days of age. In the left photo (**a**) the foal is leaning backwards with the toe slightly elevated in the typical neonatal stance with a sloping pastern, and a broken-forward hoof-pastern axis. By day 14 (**b**), the hoof is flat on the ground with a more upright hoof-pastern axis. Reproduced with permission from S.J. Curtis, The Hoof of the Horse; Newmarket Farrier Consultancy, 2019.

Changes in shape of the foal’s hoof capsule are influenced by development of the limb, conformational changes, the substrate, hoof wear, farriery and other factors. Healthy foals are usually able to stand within an hour after birth when the deciduous hoof capsule is shed and the weight-bearing tissues harden. Initially, they have poor postural control and adopt a wide-based stance to facilitate balance by increasing the dimensions of their base of support [39] and they lean backwards to relieve tension on their weak myotendinous tissues [39,40]. Leaning backwards gives the impression of a ‘broken forward’ hoof-pastern axis (Figure 9) and distributes a greater percentage of load through the heels in the forelimbs. In most foals, limb posture improves within two weeks and, by one month they appear to load the limbs normally.

Foals grow rapidly with their birth weight doubling by one month of age and tripling by three months [39,41]. As the foal grows, the hoof enlarges [23] and the hoof wall thickens to accommodate the increase in body weight. In a group of 9 Arabian foals [31], integument depth increased by approximately 4 mm from birth to 4 months of age.

**Figure 10:**
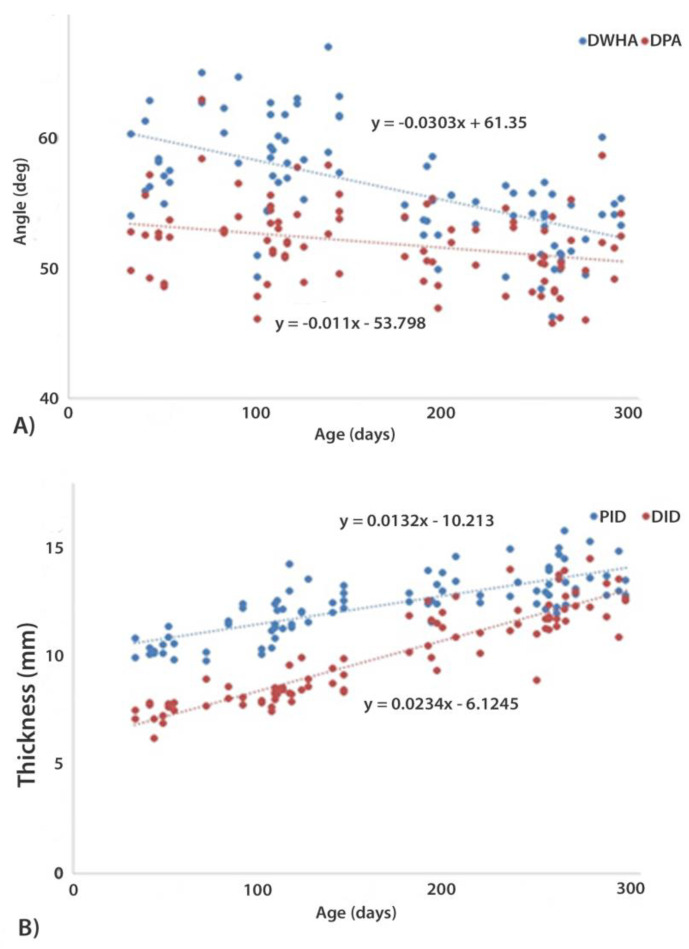
(**A**) Data points and regression lines showing convergence of dorsal hoof wall angle (DHWA) and dorsal parietal angle of the distal phalanx between 30 to 300 days of age. (**B**) Data points and regression lines for depth of the dorsal hoof wall integument proximally (PID) and distally (DID) between 30 to 300 days of age.

We investigated epidermal thickness and horn tubule characteristics in 6 Thoroughbred foals between 43–115 days. The epidermis was significantly thicker dorsally than on the medial or lateral sides (*p* < 0.05) (Table 2), though the difference was only about 1 mm. The increase in epidermal thickness and number of tubules during this time is likely to be needed to support increasing body weight. Since new tubules grow from papillae in the coronary band, epidermal thickness increases from growth at the coronary band and then as this thicker epidermis descends the hoof wall, thickness gradually increases proximo-distally. Comparing these data with radiographic measurements of integument depth from 18 Thoroughbred foals aged between 33 and 122 days, proximal integument depth ranged from 9.8 to 14.3 mm and distal integument depth ranged from 6.2 to 10.0 mm. Using regression equations (Figure 10), integument depth was calculated to increase from day 1 to day 124 by 1.6 mm proximally and 2.8 mm distally. This is smaller than the increase reported in Arabian foals in the same time period, although measurement methods are not identical [31]. Epidermal widening at the dorsum accounts for approximately half of the increase in thickness, with lamellar lengthening expected to contribute to the additional increase in integument depth. Lamellar lengthening is likely necessary to avoid developing excessive tension between the hoof wall and distal phalanx. Neither the number nor size of the horn tubules differed between regions, but tubule density was significantly greater medially and laterally. This suggests that the hoof wall of the growing foal is more malleable dorsally and has increased resistance to fracture toughness at the quarters [19].

**Figure 11:**
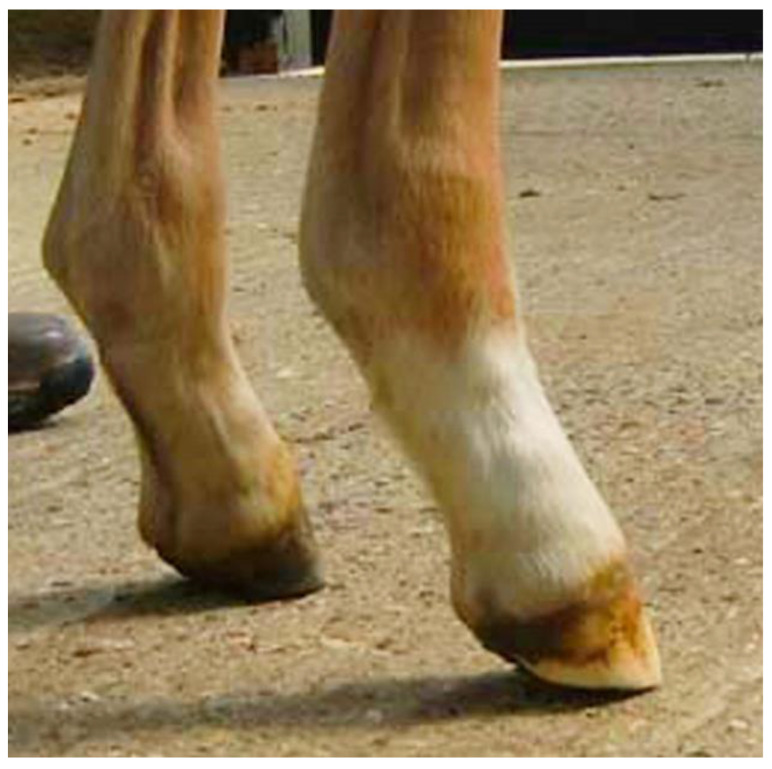
Foal showing AFD with the heels of both forelimbs elevated from the ground as a result of an inability to extend the distal interphalangeal joint. Reproduced with permission from S.J. Curtis, The Hoof of the Horse; Newmarket Farrier Consultancy, 2019.

In contrast to the normal development described above, some foals develop AFD in which the digit becomes angled more steeply and, in severe cases, the heels may even be raised from the ground (Figure 11), which suggests a dorsal shift in load distribution. The onset of this condition has been reported to be between 24–48 h [42], 1–4 months [30] and 1–8 months [43].

We investigated the veracity of our observations of foal posture and loading patterns by using a pressure mat to compare hoof loading between Thoroughbred foals with typical (*n* = 18) and AFD (*n* = 8) conformation. Load distribution was indeed significantly higher at the toe in foals showing early signs of AFD (*p* < 0.05) (Table 3). Hoof growth rate and compression rate at the dorsal hoof wall did not differ between foals with and without AFD.

### 3.3. The Foal Hoof Capsule (4–6 Months)

Thoroughbreds are reported to completely renew the fetal hoof capsule between 120 and 165 days after birth [44] and many anatomical changes occur during this period. The shape of the solear surface of the hoof has been reported to change from an oval profile in the newborn foal into a rounder profile between 4 and 6 months of age in Arabian foals [31]. Mediolateral symmetry, which is conformationally and structurally evident in the foetus and newborn foal (Table 1, Table 2 and Table 3) appears to change over this period [32].

Epidermal width in the one foal hoof that was measured in this period was 2.62 mm dorsally, 2.96 mm medially and 2.04 mm laterally. Data for hoof growth rate and hoof compression rate was limited at this age because the marks on the hoof wall were not always visible. From the data obtained for the control group, there was a trend towards a faster lateral compared to medial hoof growth rate. This was not significant, which is partly due to insufficient statistical power (F(2) = 2.061, *p* = 0.148, η^2^ = 0.137). In the control group, loading was significantly different (*p* < 0.001) between all three regions, and highly significantly different comparing medially to laterally. Widening of the solear surface together with continued evidence of greater loading medially vs. laterally (Table 3 and Table 4) may be important factors in shaping the medial and lateral quarters differently. Hoof compression rates were similar in all regions for the control group (Table 2 and Table 4). Plastic deformation was previously identified as a possible factor in shaping the hoof capsules (Dyson et al., 2011) and clearly compression of the epidermis from 0 to 6 months does occur.

### 3.4. The Foal Hoof Capsule (6–12 Months)

By 6–12 months of age, hoof length in the Thoroughbred foal is typically 95 ± 9 mm, hoof growth rate at the dorsal wall is 0.24 ± 0.08 mm/day, hoof compression rate is −0.01 ± 0.01 mm/day and the DHWA is moving closer towards the typical angles found in mature horses. During this time the shape of the hoof capsule changes from resembling an inverted cone in the foal to an obliquely truncated cone in the mature horse [19,32]. This change in shape influences the hoof-pastern axis alignment to become closer to a parallel arrangement, which agrees with reports in foals of other breeds. Reductions in DHWA have been recorded in non-Thoroughbred foals in association with a change towards a more upright dorsal parietal angle [45]. It has been suggested that these changes may involve bone absorption and bone remodeling.

The relative reductions in DHWA and dorsal parietal angle and in the proximal and distal integument depths are illustrated in Figure 10 from Thoroughbred foals measured twice at a six-month interval. Since the slope of the regression line for the DHWA was significantly steeper than that of the dorsal parietal angle (*p* < 0.001) the two surfaces became more parallel over time. The slope of the regression lines for the proximal and distal integument depths were also significantly different leading to a relatively greater increase distally and a more parallel alignment between the dorsal hoof wall and the dorsal surface of the distal phalanx. The angles were calculated to converge at 391 days when the DHWA is 49.5 deg.

Post-partum, the hoof epidermis is generated by and grows distally from the coronary band [46], which results in a proximal to distal gradient in the thickness of the epidermal hoof tissues and contributes the DHWA not being parallel with the distal phalanx. As the thicker hoof wall moves distally, the toe is projected dorsally, and this may contribute to the DHWA becoming more acute during maturation. Since the reduction of DHWA exceeded the reduction in dorsal parietal angle, the two angles approached a parallel arrangement as seen in mature horses [47].

The DHWA should also be parallel to the dorsal parietal angle of the third phalanx in mature horses and changes in the distal integument depth may be indicative of serious health concerns, such as laminar separation [48]. With this data we have demonstrated that the integument depth is wider proximally than distally in Thoroughbred foals. Therefore, this variable must be interpreted differently in foals than in adult horses and the hoof wall angle cannot be regarded as a proxy for the dorsal parietal angle of the third phalanx in foals up to approximately one year of age [45].

## 4. Conclusions

This manuscript defined structural and conformational changes in the Thoroughbred digit and hoof from late gestation to one year of age and loading patterns that may influence these changes. The data were analyzed in a chronological manner to illustrate how weightbearing and growth influence shape changes over this period.

In foetal hooves, the epidermis is thicker at the dorsum than at the quarters due to the presence of a larger number of horn tubules with a larger diameter, providing a structure with features of a mature horse to prepare for weightbearing.

In young foals, epidermal thickness at the dorsum increases, with the greater thickness being ascribed to an increase in inter-tubular horn rather than an increase in tubule number or size. Pressure within the interpapilliary space stimulates even hoof growth [49], which may account for the number and size of tubules becoming more similar between regions. The malleable hoof wall compresses evenly despite greater dorso-medial loading during standing. As the foal becomes more mobile, locomotory forces may stimulate changes in structure. For example, once the foals are able to gallop, expansion at the heels may stimulate tubular cells to develop leading to an increase in tubule density at the quarters to provide greater impact resistance [15]. The integument depth also increases during this period, with epidermal widening contributing approximately half of the overall increase, suggesting a similar increase in lamellar length. Together with a more malleable material at the dorsum due to a reduction in tubule density, lamellar lengthening is expected to assist in moderating stress during rapid skeletal growth.

At this age, some foals developed AFD with a ‘heels up’ posture that suggests a dorsal shift in loading. This study confirmed that foals with AFD have greater loading at the toe than foals with normal limb posture. As AFD can lead to club foot, the non-significant differences in hoof growth, which were due to an under powered sample, warrant further investigation. Based on these initial findings, early identification and treatment of AFD is essential for a favorable outcome.

Between 4 and 6 months of age, the foetal hoof wall is completely replaced, the width of the hoof expands and the asymmetric loading patterns in standing that have been recorded since the start of weight-bearing continue to be evident. The shape of the hoof becomes an obliquely truncated cone, which indicates that the internal stresses will now allow the hoof to function in a similar manner to the function of the hoof mechanism in mature horses. These factors may be key in changing the shape of the symmetrical foetal hoof into the asymmetrical mature hoof, but further data is required to confirm this.

Throughout the first year of life, the dorsal epidermal thickness increases at a more rapid rate distally than proximally. As a result, the DHWA decreases and becomes better aligned with the dorsal parietal angle of the distal phalanx and the hoof-pastern axis. In Thoroughbreds, parallelism of the hoof wall with the skeleton typically occurs around 391 days, which provides the weanling with an athletic hoof that optimizes functional capacity for galloping.

## Figures and Tables

**Table 1:** Mean values and (SD) for hoof wall structural variables according to region (dorsal, lateral, medial) of the hooves of 8 foetal Thoroughbreds with ages ranging from 38 days pre-partum to birth. Measurements were made in 1 mm wide section of the hoof wall in each region. Superscripts represent significant (*p* < 0.05) pairwise comparisons, ^a^ = significantly different values to the dorsal region, ^b^ = significantly different values to the lateral region, ^c^ = significantly different values to the medial region.

Variable	Dorsal	Lateral	Medial	*p*-Value
Epidermal thickness (mm)	2.84 (0.41) ^bc^	2.00 (0.44) ^a^	2.11 (0.35) ^a^	<0.001
Largest tubule diameter (mm)	0.15 (0.02) ^bc^	0.11 (0.02) ^a^	0.11 (0.02) ^a^	<0.001
Number of tubules	109 (15) ^bc^	86 (20) ^a^	86 (16) ^a^	0.019
Tubule density (tubules/mm^2^)	38.7 (5.2)	43.0 (1.2)	41.0 (4.8)	0.130

**Table 2:** Mean values and (SD) for hoof wall structural variables mid-way between the coronet and bearing surface according to region (dorsal, lateral, medial) from 6 Thoroughbred foal hooves (age 43 to 115 days). Superscripts represent significant (*p* < 0.05) pairwise comparisons, ^a^ = significantly different to values to the dorsal region, ^b^ = significantly different values to the lateral region, ^c^ = significantly different values to the medial region.

Variable	Dorsal	Lateral	Medial	*p*-Value
Epidermal thickness (mm)	4.04 (1.10) ^bc^	2.88 (0.35) ^a^	2.72 (0.20) ^a^	0.008
Largest tubule diameter (mm)	0.18 (0.04)	0.15 (0.02)	0.14 (0.02)	0.087
Number of tubules	135 (49)	121 (15)	119 (11)	0.644
Tubule density (tubules/mm^2^)	32.7 (4.7) ^bc^	42.5 (6.9) ^a^	44.2 (5.7) ^a^	0.008

**Table 3:** Mean values and (SD) for hoof growth rate, hoof compression rate and loading pattern from 18 Thoroughbred foals with typical distal limb conformation and 8 Thoroughbred foals with acquired flexural deformities (AFD). Measurements were made on two to four occasions between 0 and 4 months. Measurement for hoof growth rate and compression rate were only available from calculations made between visits. *n* = numbers of observations for each measurement. Asterisks * indicate pairs of values that differ significantly between groups (*p* < 0.05). Superscripts represent significant (*p* < 0.05) pairwise comparisons, ^a^ = significantly different to values to the dorsal region, ^b^ = significantly different values to the lateral region, ^c^ = significantly different values to the medial region.

Variable	*n*	Group	Dorsal	Lateral	Medial	*p*-Value
Hoof growth rate (mm/day)	50	Control	0.46 (0.16)	0.48 (0.20)	0.46 (0.19)	0.782
	21	AFD	0.39 (0.10)	0.51 (0.13)	0.48 (0.10)	
Hoof compression rate (mm/day)	50	Control	−0.04 (0.04)	−0.04 (0.03)	−0.05 (0.04)	0.253
	21	AFD	−0.02 (0.03)	−0.03 (0.02)	−0.03 (0.04)	
Load distribution (%)	50	Control	57.3 (16.3) *^b^	36.2 (13.6) ^ac^	63.8 (13.6) ^b^	<0.001
	21	AFD	72.2 (15.6) *	44.1 (12.2)	55.8 (12.4)	

**Table 4:** Mean values and (SD) for loading pattern from Thoroughbred foals with typical hoof and distal limb conformation compared with Thoroughbred foals with AFD conformation. Values were measured on one occasion when the foals were between 4 and 6 months. Superscripts represent significant (*p* < 0.05) pairwise comparisons, ^a^ = significantly different to values to the dorsal region, ^b^ = significantly different values to the lateral region, ^c^ = significantly different values to the medial region.

Variable	*n*	Group	Dorsal	Lateral	Medial	*p*-Value
Hoof growth rate (mm/day)	5	Control	0.41 (0.09)	0.52 (0.07)	0.40 (0.03)	0.148
	3	AFD	0.37 (0.10)	0.43 (0.06)	0.31 (0.08)	
Hoof compression rate (mm/day)	4	Control	−0.07 (0.04)	−0.07 (0.07)	−0.06 (0.06)	0.708
	3	AFD	−0.03 (0.03)	0.00 (0.01)	−0.05 (0.01)	
Load distribution (%)	11	Control	52.5 (14.9) ^bc^	37.4 (13.3) ^ac^	66.6 (13.3) ^ab^	<0.001
	3	AFD	65.0 (17.1)	36.9 (12.0)	63.1 (12.0)	

## Data Availability

Raw data is available as a Supplementary File.

## Supplementary Materials

The following supporting information can be downloaded at: https://www.mdpi.com/article/10.3390/ani12223119/s1.

## References

[1] Ryan C.T., Schaer B.L.D., Nunamaker D.M. (2006). A novel wireless data acquisition system for the measurement of hoof accelerations in the exercising horse. Equine Vet. J..

[2] Horan K., Coburn J., Kourdache K., Day P., Carnall H., Brinkley L., Harborne D., Hammond L., Peterson M., Millard S. (2022). Hoof Impact and Foot-Off Accelerations in Galloping Thoroughbred Racehorses Trialling Eight Shoe-Surface Combinations. Animals.

[3] Self Davies Z.T., Spence A.J., Wilson A.M. (2019). Ground reaction forces of overground galloping in ridden Thorough-bred racehorses. J. Exp. Biol..

[4] Mawdsley A., Kelly E.P., Smith F.H., Brophy P.O. (1996). Linear assessment of the thoroughbred horse: An approach to conformation evaluation. Equine Vet. J..

[5] van Weeren P.R., Crevier-Denoix N. (2006). Equine conformation: Clues to performance and soundness?. Equine Vet. J..

[6] Rooney J.R. (1984). The angulation of the forefoot and pastern of the horse. J. Equine Vet. Sci..

[7] O’Grady S.E. (2008). Basic farriery for the performance horse. Vet. Clin. N. Am. Equine Pract..

[8] Bramlage L.R., Auer J.A. (2006). Diagnosis, Assessment, and Treatment Strategies for Angular Limb Deformities in the Foal. Clin. Tech. Equine Pract..

[9] Witte S., Hunt R. (2009). A review of angular limb deformities. Equine Vet. Educ..

[10] Cust A.R.E., Anderson G.A., Whitton R.C., Davies H.M.S. (2013). Hoof conformation and performance in the racing Thoroughbred in Macau. Aust. Vet. J..

[11] Thomason J.J., Peterson M.L. (2008). Biomechanical and mechanical investigations of the hoof-track interface in racing horses. Vet. Clin. N. Am. Equine Pract..

[12] Pollitt C.C. (2010). The anatomy and physiology of the suspensory apparatus of the distal phalanx. Vet. Clin. N. Am. Equine Pract..

[13] Davies H.M.S. (2002). No hoof, no horse! The clinical implications of modelling the hoof capsule. Equine Vet. J..

[14] Hobbs S.J., Mather J., Rolph C., Bower J.A., Matuszewski B. (2004). In vitro measurement of internal hoof strain. Equine Vet. J..

[15] Lazarus B.S., Luu R.K., Ruiz-Pérez S., Bezerra W.B.A., Becerra-Santamaria K., Leung V., Durazo V.H.L., Jasiuk I., Barbosa J.D.V., Meyers M.A. (2022). Equine hoof wall: Structure, properties, and bioinspired designs. Acta Biomater..

[16] Huang W., Yaraghi N.A., Yang W., Velazquez-Olivera A., Li Z., Ritchie R.O., Kisailus D., Stover S.M., McKittrick J. (2019). A natural energy absorbent polymer composite: The equine hoof wall. Acta Biomater..

[17] Yoshihara E., Takahashi T., Otsuka N., Isayama T., Tomiyama T., Hiraga A., Wada S. (2010). Heel movement in horses: Comparison between glued and nailed horse shoes at different speeds. Equine Vet. J. Suppl..

[18] Goulet C., Olive J., Rossier Y., Beauchamp G. (2015). Radiographic and anatomic characteristics of dorsal hoof wall layers in nonlaminitic horses. Vet. Radiol. Ultrasound.

[19] Kasapi M.A., Gosline J.M. (1998). Exploring the possible functions of equine hoof wall tubules. Equine Vet. J. Suppl..

[20] Bragulla H. (1991). Die hinfällige Hufkapsel (Capsula ungulae decidua) des Pferdefetus und neugeborenen Fohlens. Anat. Histol. Embryol..

[21] Souza J.R.M.D., Pimentel A.M.H., Folle V.A., Pfeifer J.P.H., Schuster A.B.G., Segabinazzi L.G.T.M., Lau L.C., Martins C.F. (2017). Morphometric changes in the hoof capsule of Criollo foals from birth to weaning. Cienc. Rural.

[22] Greet T.R., Curtis S.J. (2003). Foot management in the foal and weanling. Vet. Clin. N. Am. Equine Pract..

[23] Curtis S.J. (2017). The Effect of Loading upon Hoof Wall Growth and Hoof Shape in the Thoroughbred Foal. Ph.D. Thesis.

[24] Reilly J.D., Collins S.N., Cope B.C., Hopegood L., Latham R.J. (1998). Tubule density of the stratum medium of horse hoof. Equine Vet. J. Suppl..

[25] Leach D.H. (1980). The Structure and Function of the Equine Hoof Wall. Ph.D. Thesis.

[26] Curtis S.J., Stoneham S. (1999). Effective farriery treatment of hypoflexion tendons (severe digital hyperextension) in a foal. Equine Vet. Educ..

[27] Beveridge A., Curtis S.J. (2002). Making and adapting bar shoes. Corrective Farriery: A Textbook of Remedial Horseshoeing.

[28] Butler J., Colles C.M., Dyson S.J., Kold S.E., Poulos P.W. (2013). Foot, pastern and fetlock. Clinical Radiology of the Horse.

[29] White J.M., Mellor D.J., Duz M., Lischer C.J., Voute L.C. (2008). Diagnostic accuracy of digital photography and image analysis for the measurement of foot conformation in the horse. Equine Vet. J..

[30] Curtis S.J., Rosbo-tham M., Reilly J.D. (2012). The Incidence of Acquired Flexural Deformity and Unilateral Club Foot (Uneven Feet) in Thoroughbred Foals.

[31] Faramarzi B., Salinger A., Kaneps A., Nout-Lomas Y., Greene H., Dong F. (2017). Quantitative Analysis and Develop-ment of the Fore Feet of Arabian Foals from Birth to 1 Year of Age. Vet. Comp. Orthop. Traumatol..

[32] Bidwell L.A., Bowker R.M. (2006). Evaluation of changes in architecture of the stratum internum of the hoof wall from fetal, newborn, and yearling horses. Am. J. Vet. Res..

[33] McCue P.M., Ferris R.A. (2012). Parturition, dystocia and foal survival: A retrospective study of 1047 births. Equine Vet. J. Suppl..

[34] Franciolli A.L.R., Cordeiro B.M., Da Fonseca E.T., Rodrigues M.N., Sarmento C.A.P., Ambrosio C.E., de Carvalho A.F., Miglino M.A., Silva L.A. (2011). Characteristics of the equine embryo and fetus from days 15 to 107 of pregnancy. Theriogenology.

[35] Ellis D.R., Curtis S.J. (2002). Development of the leg and foot. Corrective Farriery: A Textbook of Remedial Horseshoeing.

[36] Bragulla H. (2003). Fetal development of the segment-specific papillary body in the equine hoof. J. Morphol..

[37] Lancaster L.S., Bowker R.M., Mauer W.A. (2013). Equine hoof wall tubule density and morphology. J. Vet. Med. Sci..

[38] Roland E., Stover S.M., Hull M.L., Dorsch K. (2003). Geometric symmetry of the solar surface of hooves of thoroughbred racehorses. Am. J. Vet. Res..

[39] Nauwelaerts S., Malone S.R., Clayton H.M. (2013). Development of postural balance in foals. Vet. J..

[40] Adams R., Mayhew I.G. (1984). Neurological examination of newborn foals. Equine Vet. J..

[41] Hintz H.F., Hintz R.L., van Vleck L.D. (1979). Growth rate of thoroughbreds, effect of age of dam, year and month of birth, and sex of foal. J. Anim. Sci..

[42] Owen J.M. (1975). Abnormal flexion of the corono-pedal joint or “contracted tendons” in unweaned foals. Equine Vet. J..

[43] Fackelman G.E. (1979). Flexure deformity of the metacarpophalangeal joints in growing horses. Contin. Educ. Small Anim. Pract..

[44] Curtis S., Martin J., Hobbs S. (2014). The Hoof Renewal Time of Thoroughbred Foals from Birth. Equine Vet. J..

[45] Kroekenstoel A.M., van Heel M.C.V., van Weeren P.R., Back W. (2006). Developmental aspects of distal limb confor-mation in the horse: The potential consequences of uneven feet in foals. Equine Vet. J..

[46] Daradka M., Pollitt C.C. (2004). Epidermal cell proliferation in the equine hoof wall. Equine Vet. J..

[47] Smith S.S., Dyson S.J., Murray R.C., Weekes J. Is there an association between distal phalanx angles and deep dig-ital flexor tendon lesions?. Proceedings of the 50th Annual Convention of the American Association of Equine Practitioners.

[48] Eustace R.A. (2010). Clinical presentation, diagnosis, and prognosis of chronic laminitis in Europe. Vet. Clin. N. Am. Equine Pract..

[49] Al-Agele R., Paul E., Taylor S., Watson C., Sturrock C., Drakopoulos M., Atwood R.C., Rutland C.S., Menzies-Gow N., Knowles E. (2019). Physics of animal health: On the mechano-biology of hoof growth and form. J. R. Soc. Interface.

